# Incidence and survival in oral and pharyngeal cancers in Finland and Sweden through half century

**DOI:** 10.1186/s12885-022-09337-2

**Published:** 2022-03-02

**Authors:** Anni I. Koskinen, Otto Hemminki, Asta Försti, Kari Hemminki

**Affiliations:** 1 Department of Otorhinolaryngology- Head and Neck Surgery, Helsinki University Hospital and University of Helsinki, PO Box 263, 00029 Helsinki, Finland; 2 Department of Urology, Helsinki University Hospital and University of Helsinki, Helsinki, Finland; 3 Cancer Gene Therapy Group, Translational Immunology Research Program, University of Helsinki, Helsinki, Finland; 4 Hopp Children’s Cancer Center (KiTZ), Heidelberg, Germany; 5 Division of Pediatric Neurooncology, German Cancer Research Center (DKFZ), German Cancer Consortium (DKTK), Heidelberg, Germany; 6 Faculty of Medicine and Biomedical Center in Pilsen, Charles University in Prague, 30605 Pilsen, Czech Republic; 7 Division of Cancer Epidemiology, German Cancer Research Centre (DKFZ), 69120 Heidelberg, Germany

**Keywords:** Oral cancer, Pharyngeal cancer, Human papilloma virus, Smoking, Alcohol

## Abstract

**Background:**

Cancers of the oral cavity and pharynx encompass a heterogeneous group of cancers for which known risk factors include smoking, alcohol consumption and human papilloma virus (HPV) infection but their influence is site-specific with HPV mainly influencing oropharyngeal cancer. Their incidence and survival rates are not well known over extended periods of time.

**Patients/methods:**

Data were obtained for Finnish (FI) and Swedish (SE) patients from the Nordcan database recently updated through 2019. Age-adjusted incidence trends (FI from 1953, SE from 1960) and relative survival rates for years 1970 through 2019 were calculated.

**Results:**

We observed a prominent increase in oral and oropharyngeal cancers in FI and SE men and women but the trend for oral cancer was interrupted for SE men in 1985 and possibly also for FI and SE women in 2015. The trend changes in male and female oral cancer was confirmed in data for Denmark and Norway. Relative survival for these cancers has improved overall but they differed for one cluster of oral, oropharyngeal and nasopharyngeal cancers with 60–70% 5-year survival in the last period and hypopharyngeal cancer with 25% male survival. In all these cancers, survival for old patients was unfavorable.

**Discussion/conclusion:**

We hypothesize that reduction in smoking prevalence helped to stop the increase in oral cancer especially in men. As the prevalence of smoking is decreasing, HPV is becoming a dominant risk factor, particularly for the increasing oropharyngeal cancer. Prevention needs to emphasize sexual hygiene and HPV vaccination.

**Supplementary Information:**

The online version contains supplementary material available at 10.1186/s12885-022-09337-2.

## Introduction

Cancers of the oral cavity and pharynx are a part of head and neck cancers and include, according to International Classification of Disease (ICD) version 10, cancers in the oral cavity, oropharynx, nasopharynx and hypopharynx; these are predominantly of squamous cell carcinoma histology [1]. According to the Swedish and Danish Cancer Registries the case ranking follows the frequency order—oral, oropharyngeal, hypopharyngeal and nasopharyngeal cancers, with male cases outnumbering female cases [2, 3]. The main risk factors for these cancers in Western countries are tobacco, alcohol and their interactions and infection with human papillomavirus (HPV), but their contributions may vary by site [1, 4–6]. According to the International Agency for Research on Cancer (IARC), nasopharyngeal cancer may be less responsive to tobacco carcinogenesis than other pharyngeal or oral cavity cancers [4]. Smoking cessation decreases the risk compared to continuing smoking and after 10 years of cessation oral cancer incidence may be at the level of non-smokers. While smoking and alcohol interact at these cancer sites, moderate consumption of alcohol by non-smokers may not be a strong risk factor for nasopharyngeal cancer [4]. According to IARC, HPV is associated with oral and oropharyngeal cancers [5]. However, the etiological role of HPV is much more prominent in oropharyngeal than oral cancers [7, 8]. The incidence of oropharyngeal and oral cancers has been increasing in Western countries during the recent decades which has been associated with HPV infection and oral sex [2, 9–11]. Oral HPV infection has been shown to be the primary risk factor for HPV-related oropharyngeal cancer, and it is estimated that over 90% of oral HPV infections are sexually acquired [12, 13]. Head and neck cancers are increased in immunocompromised individuals, such as kidney transplant patients, which may imply viral activation or other sensitivity to immune disturbance [14]. These mechanisms may also explain high risks of any head and neck cancer as second primary cancers after a number of first primary cancers [15, 16]. Head and neck cancers may present as multiple synchronous or metachronous primary tumors, referred to as ‘field cancerization’ [1].

HPV-positive and -negative head and neck cancers differ from each other in many ways including prognosis [1, 2, 17–19]. Since HPV-positive oropharyngel cancer is shown to be more responsive to oncologic treatments and have a better prognosis, it was distinguished in the tumor-node-metastasis (TNM) classification distinct from HPV-negative cancer in the 8th Edition of the American Joint Committee on Cancer (AJCC) Cancer Staging Manual [20, 21]. In general in oral and pharyngeal cancer, the treatment approach to every individual patient is guided by anatomical subsite, stage, disease and patient characteristics, functional considerations and patient wishes. HPV status is considered but has currently no dramatic effect on how patients are treated. Treatment modalities are surgery, radiation or chemoradiation or their combinations [1].

In a series of Danish studies, incidence and survival in specific head and neck cancers were studied up to year 2014 [2, 22–25]. The highest increase in incidence was noted for oropharyngeal cancers but also hypopharyngeal cancer was increasing; oropharyngeal cancer showed evidence of a birth-cohort effect, a low risk in those born in period 1925 to 1935, which was interpreted as the war time movement restrictions and curfews limiting human contacts [22]. Co-morbidities were common in head and neck cancer patients and these contributed to worsened survival [26].

We report here result on incidence and survival in oral and pharyngeal cancers in Finland (FI) and Sweden (SE) over a half century. The neighboring countries FI and SE are historically and culturally related but have had many differences in economic resources which has influenced health and social policy. FI has been looking up to SE in the organization of medical care, yet with lower resources reflecting in health care expenditure (www.macrotrends.net). Both countries have offered medical care practically free-of-charge to the population, thus the present results provide a genuine ‘real world’ experience of medical outcomes in these cancers. A further point relevant to these cancers is the extensive differences between smoking histories in these two countries, with FI men being among the heaviest smokers after World War II and SE men emerging as non-smoking champions (www.pnlee.co.uk/ISS.htm) [27].

## Materials and methods

The data used originate from the Nordcan database 2.0 which is a compilation of data from the Nordic cancer registries [28]. These registries are presented in detail by Pukkala and coworkers [29]. The database was accessed at the International Agency for Cancer (IARC) website (https://nordcan.iarc.fr/en/database#bloc2). Because Nordcan 2.0 does not enable age-specific survival analyses these were done in the earlier version of Nordcan including data to the end of 2016 (https://www-dep.iarc.fr/NORDCAN/english/frame.asp). Coverage of cancers in the FI and SE cancer registries is generally considered high [29]. The SE cancer registry does not consider cancers in death notifications and some 4% cases may be missed because of this; an overall comparison of various health records showed that the coverage was over 90% [29, 30]. Oral cavity cancer includes IDC-10 codes C00.3-C00.5, C02-C04 C05.0, C05.8-C05.9, C06. Pharyngeal cancers are defined according to ICD-10 as cancers of the oropharynx (C01, C05.1-C05.2, C09, C10.0, C10.2-C10.9, C14.0, C14.2-C14.8), nasopharynx (C11) and hypopharynx (C12-13); we did not include pharynx, ill-defined (C14) because of small case numbers. Histology of these cancers is squamous cell carcinoma (~ 90%) and the other larger entity is undifferentiated cancer (probably originating from squamous cell carcinoma). FI and SE cancer registries classify lymphomas into an independent entity, irrespective of localization.

Data on FI and SE patients were extracted from Nordcan and the follow-up was extended until the end of 2019. For incidence data, the starting date was the earliest available, 1953 for FI, 1960 for SE. For age standardization the world standard population was used. Survival data for relative survival were available from 1970 onwards and the analysis was based on the cohort survival method for the first four 10-year periods, and a hybrid analysis combining period and cohort survival in the last period 2010–2019, as detailed [31]. Age groups 0 to 89 were considered, and for age-standardization the International Cancer Survival Standard was used. The FI and SE life tables were used to calculate the expected survival. In some incidence trend analysis also data from Denmark (DK) and Norway (NO) were included.

In graphic presentation of incidence rates, lines were smoothed by the LOESS regression algorithm (bandwidth: 0.1, indicating the proportion of all data points contributing to each shown value). In some trend analysis estimated annual percent change (EAPC) and its 95% confidence interval (CI) were assessed.

## Results

In period 1970 to 2019, the patient numbers, incidence, cumulative incidence and median age at diagnosis of oral and pharyngeal cancers were collected from the Nordcan database (Table 1). Oral cavity cancer was the most common site-specific cancer in both FI and SE men (2.4 and 2.1/100,000) and women (1.5 and 1.4/100,000). Cumulative incidence (0–74 years) was 0.28 and 0.25%, respectively for men and 0.17 and 0.15% for women. Nasopharyngeal cancer was the rarest male cancer while for women it was equally rare than hypopharyngeal cancer.. The median diagnostic ages were in the low 60 s for oropharyngeal and nasopharyngeal cancers but they were around 70 year for SE oral and hypopharyngeal cancers and for FI women.

**Table 1:** Case numbers, age-standardized (world) incidence/100,000, cumulative incidence (%, 0–74 y) and median age at diagnosis of oral and pharyngeal cancer patients in Finland and Sweden, 1970–2019

FINLAND MEN					SWEDEN MEN			
Cancer	Number	Incidence	Cum Incidence	Median age	Number	Incidence	Cum Incidence	Median age
Oral Cavity	4243	2.4	0.28	63	7780	2.1	0.25	67
Oropharynx	2519	1.4	0.18	61	6389	1.9	0.23	62
Nasopharynx	481	0.29	0.03	61	1285	0.41	0.04	60
Hypopharynx	993	0.53	0.07	66	2467	0.63	0.08	68
FINLAND WOMEN					SWEDEN WOMEN			
Cancer	Number	Incidence	Cum Incidence	Median age	Number	Incidence	Cum Incidence	Median age
Oral Cavity	3913	1.5	0.17	71	6417	1.4	0.15	73
Oropharynx	998	0.46	0.05	60	2609	0.71	0.09	63
Nasopharynx	281	0.14	0.02	64	637	0.18	0.02	65
Hypopharynx	331	0.14	0.02	69	735	0.16	0.02	70

**Fig. 1:**
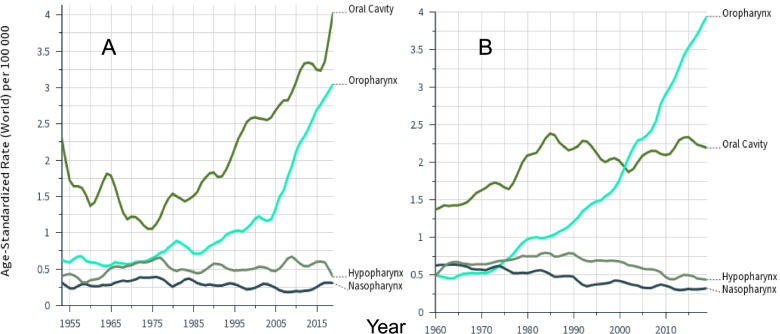
Age-standardized incidence in cancers of the oral cavity and pharynx among Finnish (**A**) and Swedish (**B**) men through 2016 based on the Nordcan database. Note that the follow-up started in 1953 in Finland and in 1960 in Sweden. Smoothing bandwidth of 0.1

Incidence rates for oral and pharyngeal cancers in FI (A) and SE men (B) are shown in Fig. 1. FI incidence data were recorded since 1953 and showed an early peak of oral cancer. Remarkably, the only cancers with increases in incidence were oral and oropharyngeal cancers, starting in the 1970. Increase of oral cancers stalled in 1985 for SE men, and was crossed by the incidence curve of oropharyngeal cancer after year 2000. Incidence in hypopharyngeal and nasopharyngeal cancers was stable in FI and declined in SE.

**Fig. 2:**
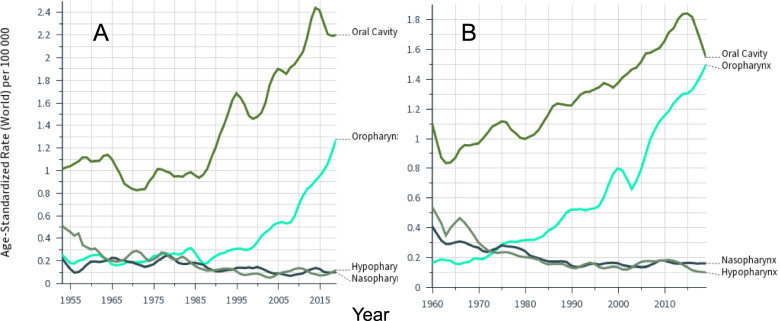
Age-standardized incidence in cancers of the oral cavity and pharynx among Finnish (**A**) and Swedish (**B**) women through 2016 based on the Nordcan database. Note that the follow-up started in 1953 in Finland and in 1960 in Sweden. Note the different scale of Y axis. Smoothing bandwidth of 0.1

The patterns were similar in female cancers (Fig. 2). Oral cancer incidence was higher in FI compared to SE while for oropharyngeal cancer the opposite was true. The incidence in female hypopharyngeal and nasopharyngeal cancers declined throughout the follow-up time. For oral cancer, an incidence maximum was noted at around 2015 and we tested the significance of these incidence changes using EAPC. For FI in years 2006–9 EAPC showed an increase of 3.55% (-8.22 to 16.52%), in years 2010–14 of 1.65% (-26.95 to 41.43%) and in years 2015–19 a decrease of – 1.94% (-28.12 to 33.77%). For SE EAPCs were 2.44% (-10.14 to 16.79%), 0.69% (-30.66 to 44.95%) and -2.44% (-30.40 to 36.75%). Thus none of these changes were significant.

Trends in the cancer incidence of the oral cavity were analyzed in all four Nordic countries (Supplementary Fig. 1). For DK and NO men, the incidence culminated before year 2000 and 1990, respectively. Thus only the increasing trend for FI men deviated from that of the other countries. For women, the trend turned into decline in all countries at around 2015.

**Fig. 3:**
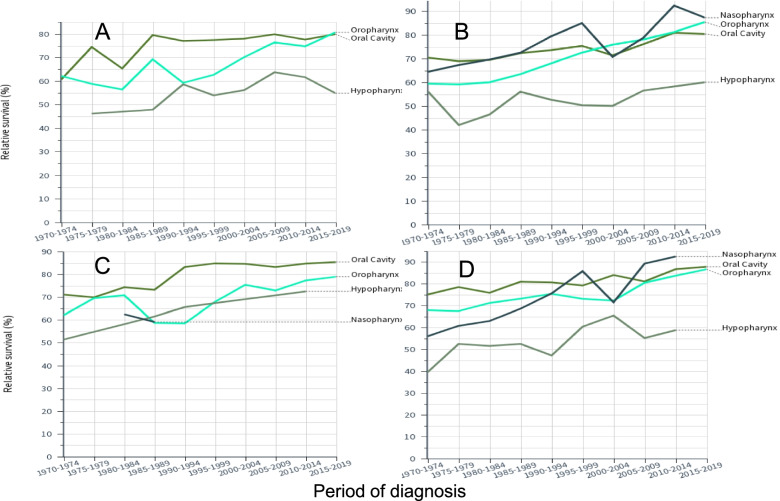
Relative 1-year survival in Finnish men (**A**) and women (**C**) and in Swedish men (**B**) and women (**D**). Confidence intervals of the datapoints can be found in Tables 2 and 3. Some curves are missing or incomplete because of low case numbers. Note the different scale of Y axis in (**A**)

Relative 1-year survival data for oral and pharyngeal cancers are plotted in Fig. 3 (A, FI men, B, SE men, C, FI women and D, SE women; nasopharyngeal cancer curves were missing for FI because of low case numbers). The plots illustrate the similar starting levels in 1970–79 and improvement over the 50-year period. SE survival rates for oropharyngeal and nasopharyngeal cancers were at around 85–90% in 2010–19 while FI rates were close to 80%r. For SE male and female hypopharyngeal cancers the initial survival was some 20% units below the other cancers and the gap widened with time.

**Fig. 4:**
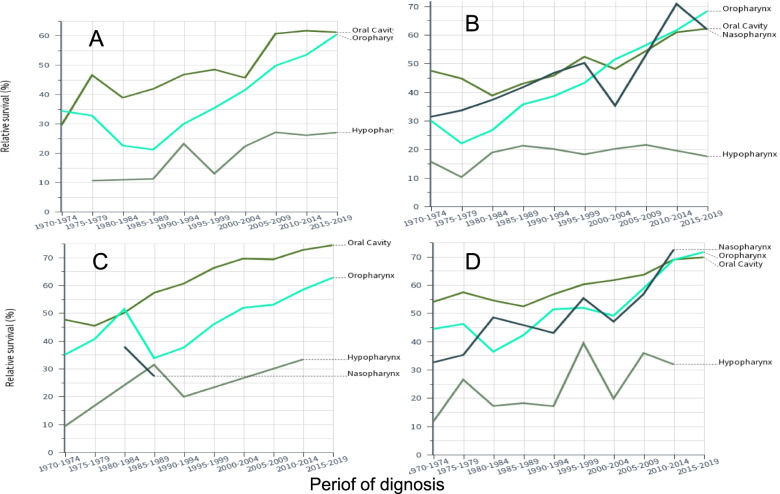
Relative 5-year survival in Finnish men (**A**) and women (**C**) and in Swedish men (**B**) and women (**D**). Confidence intervals of the datapoints can be found in Tables 2 and 3. Some curves are missing or incomplete because of low case numbers. Note the different scale of Y axis in (**A**)

Relative 5-year survival rates are shown in Fig. 4. Survival increased for oral, oropharyngeal and nasopharyngeal cancers from 30–40% to 60–70%, Survival in hypopharyngeal cancer was far lower, particularly for SE men.

We show detailed relative 1-year and 5-year survival rates for oral and pharyngeal cancers for FI and SE men in 10-year periods in Table 2. We marked the periods (*) when survival increased significantly (95% confidence intervals (CIs) did not overlap) and bolded the last survival figures when it was significantly improved compared to the first survival figures. Underlining was used for significant difference between FI and SE. Survival for oral and oropharyngeal cancers (both 1-year and 5-year) improved in both countries (significant increase between the last and first periods). In the last period SE 1-year survival for oropharyngeal cancer was significantly higher than the FI one (84.6 vs. 78.1%). For SE also nasopharyngeal cancer 1-year and 5-year survival increased significantly over the study period, as did 5-year survival for SE hypopharyngeal cancer (yet with survival of 22.3% showing the lowest final rate recorded). Most significant periodic increases were noted towards the end of follow-up. Of note, statistical significance depends on case numbers and thus common events reach significance levels at relatively small differences in point estimates.

**Table 2:** Relative 1- and 5-year survival (% and 95%CI) in cancers of the oral cavity and pharynx in FI and SE men

**FI men 1-y**	**1970–1979**	**1980–1989**	**1990–1999**	**2000–2009**	**2010–2019**
Oral Cavity	69.6 [62.7–75.5]	72.7 [67.1–77.4]	77.2 [73.0–80.8]	79.7 [76.6–82.5]	**79.0 [76.5–81.2]**
Oropharynx	61.4 [51.9–69.5]	62.3 [54.1–69.4]	60.9 [52.5–68.2]	75.8*[69.9–80.7]	**78.1 [74.9–81.0]**
Nasopharynx	66.4 [50.6–78.2]	74.4 [59.2–84.6]	67.8 [54.3–78.0]	73.8 [59.8–83.5]	71.7 [57.4–81.9]
Hypopharynx	47.1 [37.5–56.1]	48.4 [39.0–57.1]	58.0 [49.1–65.9]	60.6 [53.1–67.3]	59.9 [53.5–65.8]
**SE men 1-y**	**1970–1979**	**1980–1989**	**1990–1999**	**2000–2009**	**2010–2019**
Oral Cavity	69.7 [66.6–72.5]	71.1 [68.4–73.6]	74.5 [71.9–76.8]	73.5 [70.9–75.8]	80.8*[78.8–82.6]
Oropharynx	59.5 [54.0–64.5]	62.2 [57.9–66.2]	70.5*[66.7–74.0]	77.0 [73.9–79.7]	84.6*[82.7–86.4]
Nasopharynx	67.6 [60.4–73.7]	71.6 [64.8–77.4]	82.4 [74.9–87.9]	74.2 [64.9–81.4]	**88.4 [80.3–93.4]**
Hypopharynx	50.4 [45.3–55.3]	50.6 [46.1–54.9]	52.5 [47.7–57.0]	53.8 [49.1–58.4]	58.0 [52.4–63.1]
**FI men 5-y**	**1970–1979**	**1980–1989**	**1990–1999**	**2000–2009**	**2010–2019**
Oral cavity	38.7 [30.2–47.2]	41.2 [34.4–47.9]	48.4 [42.9–53.7]	55.9 [51.6–60.0]	**61.2 [57.3–64.8]**
Oropharynx	32.2 [22.7–42.0]	23.0 [16.3–30.5]	33.3 [25.1–41.6]	47.1 [40.4–53.5]	59.1*[54.3–63.6]
Nasopharynx	35.0 [21.5–48.8]	46.1 [29.4–61.3]	36.8 [23.4–50.2]	51.0 [35.7–64.4]	49.9 [33.8–64.1]
Hypopharynx	11.8 [5.7–20.3]	10.8 [5.6–17.9]	17.2 [10.6–25.1]	26.0 [19.4–33.0]	25.7 [19.2–32.6]
**SE men 5-y**	**1970–1979**	**1980–1989**	**1990–1999**	**2000–2009**	**2010–2019**
Oral cavity	45.5 [41.6–49.4]	41.4 [38.1–44.6]	48.7*[45.4–52.0]	51.0 [47.8–54.1]	61.8*[58.7–64.8]
Oropharynx	24.3 [19.2–29.7]	31.1 [26.7–35.6]	41.6*[37.2–45.9]	53.8*[50.0–57.4]	66.2*[63.2–69.1]
Nasopharynx	33.9 [26.6–41.4]	39.6 [32.3–46.8]	48.9 [38.9–58.1]	43.4 [33.5–52.9]	**61.8 [49.9–71.7]**
Hypopharynx	12.2 [8.8–16.0]	18.5 [14.9–22.4]	19.1 [15.4–23.1]	20.0 [16.1–24.2]	**22.3 [17.2–27.8]**

Bolding: Survival significantly increased between last and first period. ^*^Survival significantly increased between the periods. Underlining: Significant country difference

Similar analysis of female survival is shown in Table 3, and the results are quite similar to men. Overall the final survival figures tended to be higher than the male ones, and all oral cancer survival rates were significantly higher than the male rates. The highest survival recorded in this study was 92.0% for 1-year survival in SE women with nasopharyngeal cancer. No significant country differences were noted.

**Table 3:** Relative 1- and 5-year survival (% and 95%CI) in cancers of the oral cavity and pharynx in FI and SE women

**FI women 1-y**	**1970–1979**	**1980–1989**	**1990–1999**	**2000–2009**	**2010–2019**
Oral Cavity	70.5 [64.8–75.5]	73.9 [69.2–77.9]	83.6*[80.4–86.3]	82.9 [80.2–85.3]	**84.8 [82.5–86.8]**
Oropharynx	66.8 [54.9–76.2]	66.5 [56.1–74.9]	63.3 [52.1–72.6]	74.1 [64.4–81.6]	79.8 [74.5–84.1]
Nasopharynx	53.6 [38.7–66.4]	62.7 [48.3–74.1]	71.0 [55.5–81.9]	80.6 [56.8–92.1]	82.2 [61.4–92.4]
Hypopharynx	50.2 [39.6–59.9]	60.8 [47.9–71.4]	56.9 [42.3–69.1]	55.6 [39.3–69.2]	65.2 [49.1–77.3]
**SE women 1-y**	**1970–1979**	**1980–1989**	**1990–1999**	**2000–2009**	**2010–2019**
Oral Cavity	76.4 [73.2–79.3]	77.6 [74.7–80.3]	79.3 [76.7–81.7]	81.3 [79.0–83.4]	86.5*[84.6–88.1]
Oropharynx	66.7 [59.1–73.2]	72.2 [65.8–77.6]	74.9 [69.8–79.3]	76.7 [72.1–80.6]	86.2*[83.2–88.6]
Nasopharynx	58.8 [50.8–65.9]	66.5 [57.4–74.0]	80.5 [69.6–87.9]	76.4 [64.1–84.9]	92.0*[79.0–97.1]
Hypopharynx	46.2 [38.5–53.5]	50.5 [41.7–58.7]	50.5 [41.1–59.2]	57.7 [48.3–65.9]	61.3 [52.1–69.3]
**FI women 5-y**	**1970–1979**	**1980–1989**	**1990–1999**	**2000–2009**	**2010–2019**
Oral Cavity	46.4 [39.7–52.8]	53.9 [48.2–59.3]	63.4 [58.8–67.7]	68.3 [64.5–71.8]	**73.3 [69.6–76.6]**
Oropharynx	38.3 [26.2–50.2]	44.9 [34.0–55.2]	41.1 [29.4–52.4]	52.2 [40.5–62.6]	**62.6 [55.4–68.9]**
Nasopharynx	29.9 [17.5–43.3]	35.8 [22.8–49.0]	53.8 [37.2–67.8]	46.1 [24.4–65.3]	**75.1 [54.4–87.4]**
Hypopharynx	12.6 [6.5–20.7]	18.5 [9.5–29.9]	20.3 [10.2–32.8]	21.5 [10.0–35.8]	30.3 [15.1–47.0]
**SE women 5-y**	**1970–1979**	**1980–1989**	**1990–1999**	**2000–2009**	**2010–2019**
Oral Cavity	55.6 [51.3–59.7]	52.4 [48.6–56.1]	58.2 [54.7–61.6]	62.2 [59.0–65.2]	68.7*[65.6–71.7]
Oropharynx	44.5 [35.9–52.7]	39.4 [32.5–46.2]	52.4*[46.3–58.1]	55.0 [49.5–60.1]	69.9*[65.1–74.2]
Nasopharynx	34.4 [26.4–42.5]	47.1 [37.3–56.3]	50.1 [38.0–61.0]	51.7 [37.0–64.6]	**67.2 [48.7–80.2]**
Hypopharynx	17.6 [11.8–24.3]	13.8 [8.4–20.6]	26.9 [18.6–35.9]	27.6 [19.6–36.2]	**34.7 [25.0–44.6]**

Bolding: Survival significantly increased between last and first period. ^*^Survival significantly increased between the periods

Synthesis of Table 2 and 3 shows improvements in survival between 1970–9 and 2010–9 for oral cancer (combining FI and SE males and females) with 11.2% units in 1-year survival and 19.9% units in 5-year survival; for oropharyngeal cancer these were 18.7/29.6% units; for nasopharyngeal cancer these were 22.0/30.2% units; for hypopharyngeal cancer these were 12.8/10.9% units. One can also calculate the difference in survival between years 1 and 5 as an indication if survival improved between years 1 and 5. The difference for men and women for oral and oropharyngeal cancer in 1970–79 was about 28% unit and in 2010–19 it was about 16% units; thus survival improved between years 1 and 5 after diagnosis. For nasopharyngeal and hypopharyngeal cancer the differences were about 31 and 26% units, respectively. Thus survival increased only modestly between years 1 and 5.

Age-groups specific survival was analyzed in period 2012–2016 based on the previous version of the Nordcan database (Table 4). Both 1- and 5-survival declined with diagnostic age.. For oropharyngeal cancer 1-year survival for the oldest, 80–89 year old was about 1/3 lower than that of the youngest patients but male 5-year survival was only less than half. For nasopharyngeal cancer the age dependent decrease in survival was less than for oropharyngeal cancer.

**Table 4:** Survival % (1-year/5-year) in age groups of oral and pharyngeal cancers diagnosed in 2012–16

**Male**	**0–49**	**50–59**	**60–69**	**70–79**	**80–89**
Oral cavity					
Finland	86/75	82/61	82/57	73/60	72/50
Sweden	95/77	89/66	80/55	77/50	60/47
Oropharynx					
Finland	95/80	85/68	80/60	66/41	66/41
Sweden	96/91	94/78	88/67	77/52	61/36
Nasopharynx					
Finland	93/42	93/42	71/51	71/51	71/51
Sweden	92/73	100/66	97/65	91/55	91/55
Hypopharynx					
Finland	61/27	61/27	63/28	53/23	53/23
Sweden	62/31	62/31	63/26	62/21	32/0
**Female**					
Oral cavity					
Finland	87/77	91/78	90/75	82/69	69/54
Sweden	97/88	88/68	89/69	85/64	74/47
Oropharynx					
Finland	96/77	96/77	86/70	64/43	64/43
Sweden	100/87	96/80	89/71	74/52	68/51
Nasopharynx					
Finland	94/88	94/88	85/73	85/73	85/73
Sweden	97/90	97/90	100/85	94/46	94/46

Hypopharyngeal data were not shown for women because of low case numbers

## Discussion

The recently updated Nordcan data through year 2019 show the dramatic increase in the incidence of oropharyngeal cancer which started in SE men in the 1970s and jumped eightfold by 2019; for FI men the increase started a decade later and jumped fourfold. The female incidence increased in parallel but at some 40% of the male incidence. Cancer of the oral cavity was increasing even at a higher level but at a slower rate than oropharyngeal cancer. However, the novel finding was the stagnation of SE male oral cancer incidence in 1985 which was also found for DK and NO men. We observed a possible trend change for female oral cancer in all Nordic countries at around 2015 but further years will show if the change is real. The rates for the other pharyngeal cancers remained constant or declined. As to survival, improvements were noted for all cancers but hypopharyngeal cancer survival remained poor. For all cancers, the concern in survival was the age-related disadvantage. We try to put these findings into perspective below.

What happened to SE male oral cancer rates around 1985 when the increasing trend abruptly sifted, opposite to the constantly increasing trends in this cancer in other groups? Alcohol drinking and tobacco smoking have strong synergistic effects on oral cancer [4]. FI men were heavy smokers after World War II and their smoking prevalence remained at 60% until 1960 at a level higher than that for SE men (www.pnlee.co.uk/ISS.htm). The early incidence peak for FI male oral cancer (Fig. 1A) is most likely due to the high historical smoking prevalence. Smoking prevalence declined fast for SE men [32]; they reduced their daily smoking prevalence from 28.4% in 1988/89 to 15.7% in 2004/05 to the lowest level among European men, while among FI men 35.5% smoked in 1988/89 and 28.0% smoked in 2004/05 [33]. SE men compensated tobacco smoking by oral tobacco (snuff/snus) but this habit seems not to cause oral cancer [27, 33–35]. Among SE women smoking prevalence slowly decreased to about 20% in 2000 matching prevalence of FI women for whom the prevalence had slowly increased. After 2000, female smoking prevalence slowly decreased in both countries [36]. The incidence in lung cancer in SE women exceeded that of SE men after 2010 which was an epochal incidence crossing in the history of tobacco carcinogenesis [27, 35]. We hypothesize that the reduction in smoking level and modest consumption of alcohol among SE men helped to stop the increasing trend in cancer of the oral cavity [36]. Recent reduction in smoking prevalence may contribute to the culmination of oral cancer incidence in FI and SE women. Why this is not seen among FI men may be due to a continued overuse of alcohol [36]; FI men have had high death rate due to alcoholic liver disease, exceeding SE men about fivefold [37]. Finally, why is the incidence in oropharyngeal cancer still increasing as it also shows synergy with alcohol and smoking [4]. The reason may be its heavy etiological dependence on HPV for with prevalence has been increasing [7, 8].

As these cancers are rare, the survival curves show large fluctuations. Yet the survival curves appeared to cluster oral, oropharyngeal and nasopharyngeal cancers in one group and hypopharyngeal cancer in another other group, much below the others. In terms of absolute increase in survival % in the 50 year period, the gain in hypopharyngeal cancer was the weakest, 12.8/10.9% units (1-year/5-year survival). Improvement in oral cancer was not much better, 11.2 and 19.9% units. Oropharyngeal cancer (18.7/29.6% units) and nasopharyngeal cancer (22.0/30.2% units) were the winners in absolute survival gains. It is known that survival is better for HPV-infected cases of oropharyngeal cancer and the infection rates have increased over time. A large study in the Stockholm area reported HPV positivity in oropharyngeal cancer at 70%, slightly higher for men than for women (72 vs 64%) and a marginal increase from 67 to 70% over the period from 2000 through 2016 [11]. Recent results from the Nordic countries, including FI and SE, showed that HPV infection in oropharyngeal cancer among middle-aged men had tripled in 30 years [38]. Thus it is likely that increasingly higher proportion of HPV-infected patients are driving the increasing survival rates for oropharyngeal cancer. The present results showed no large differences in oropharyngeal cancer survival between men and women. In line with increasing prevalence of HPV infections, survival of the old patients, particularly old men, was much poorer than that of younger patients of whom a larger proportion were assumedly HPV positive.

If HPV infections have contributed to the positive development in oropharyngeal cancer survival then other factors have advanced survival in the other cancers. These are likely to include earlier detection because of improved imaging and more efficient treatment, including radiotherapy [2, 24, 25]. Particularly, utilization of positron emission tomography (PET), has led to earlier diagnosis and more precise staging in these cancers [39, 40]. Standardized treatment protocols, multidisciplinary tumor boards together with advances in surgical and oncological treatments have also contributed to increased survival [41, 42]. The difference in survival between years 1 and 5 is an indicator of survival improvement between years 1 and 5. The difference for men and women for oral and oropharyngeal cancer in 1970–79 was about 28% unit and in 2010–19 it was about 16% units indicating survival improvements between years 1 and 5 after diagnosis. For nasopharyngeal and hypopharyngeal cancer the difference was only 5% units indicating only a modest improvement between years 1 and 5 compared to 1-year survival increase. One can assume that patients surviving 5 years may have a good chance of being cured, and this varied for 60 to 70% for all but hypopharyngeal cancer for which male survival was barely 25%. Time to cure is dependent on diagnostic age and for cancer of the oral cancity and pharynx it varies from about 7 years at age 15–44 years to about 17 at age 65–74 [43].

The main limitations of this study are lacking data on detailed clinical characteristics of the patients and their risk factors which imply that all inferences are ecological. Due to the rare cancers under study many case numbers were small and the results were subject to random variation. As smoking is an important risks factor, the present populations offer historically a large contrast with heavy smoking FI men and more recently the lowest European smoking levels among SE men [32, 44]. At the low background level of smoking the role of HPV infections or other risk factors may be emerging. The strengths were long-term follow-up of high level cancer registry data including a recent up-dating to the end of 2019. Nation-wide coverage is an important guarantee against selection bias and for providing a true ‘real world’ view onto cancer reality at the population level.

In conclusion, using the Nordcan data we could confirm the prominent increase in oropharyngeal cancer in FI and SE men and women which however paralleled with even higher levels of oral cancer. Among SE men the rising incidence of oral cancer was interrupted in 1985 and thereafter followed an even trend. We hypothesize that reduction in smoking prevalence helped stop the increase. We could report culminations of FI and SE female rates for oral cancer in 2015, and we assume that reduction in smoking prevalence is also contributing to this trend change; yet further follow-up is needed to confirm the trend change. Relative survival for these cancers has improved overall but they appeared to cluster in a group of oral, oropharyngeal and nasopharyngeal cancers with 60–70% 5-year survival in the last period and hypopharyngeal cancer with 25% male survival. In all these cancers, old patients survived less favorably than young patients which may be related to the presence of comorbidities and needs to be to be addressed in the clinical setting. For the rapidly increasing oropharyngeal cancer, HPV infection is the leading current cause. Efficient prevention should benefit if the routes of HPV infections to non-genital organs were better characterized, which in addition to oral sex, may include (deep) kissing, self-infection from genital HPV, mother-newborn transmission etc. [45, 46]. Although gender neutral HPV vaccination will most likely turn down the growing incidence curves, this is assumed to take several decades to show population effect [38, 47–49]. In the Nordic countries vaccination targeted birth cohorts from 1995 onwards but some 50 older birth cohorts are at risk and would benefit from information campaigns on sexual hygiene and development of screening programs for detection of precursor lesions for oropharyngeal cancer [38].

## Supplementary Information

**Additional file1: Figure 1.** Incidence trends in cancer of the oral cavity in the Noridic countries, A males, B females.

## Data Availability

Publically available NORDCAN data can be accessed at (https://NORDCAN.iarc.fr/en/database#bloc2).

## Abbreviations

AJCC: American Joint Committee on Cancer
CI: Confidence interval
DK: Denmark
EAPC: Estimated annual percent change
FI: Finland
HPV: Human papilloma virus
IARC: International Agency for Research on Cancer
ICD: International classification of diseaases
NO: Norway
SE: Sweden
TNM: Tumor-node-metastasis
