# Supplementary material for: Incidence and survival in oral and pharyngeal cancers in Finland and Sweden through half century
Source: BMC Cancer. 2022 Mar 2;22:227. doi: 10.1186/s12885-022-09337-2 (PMC8889707; doi:10.1186/s12885-022-09337-2)
Supplement: Supplementary file 1 — Additional file1: Figure 1. Incidence trends in cancer of the oral cavity in the Noridic countries, A males, B females. [file 12885_2022_9337_MOESM1_ESM.pptx]

## Slide 1
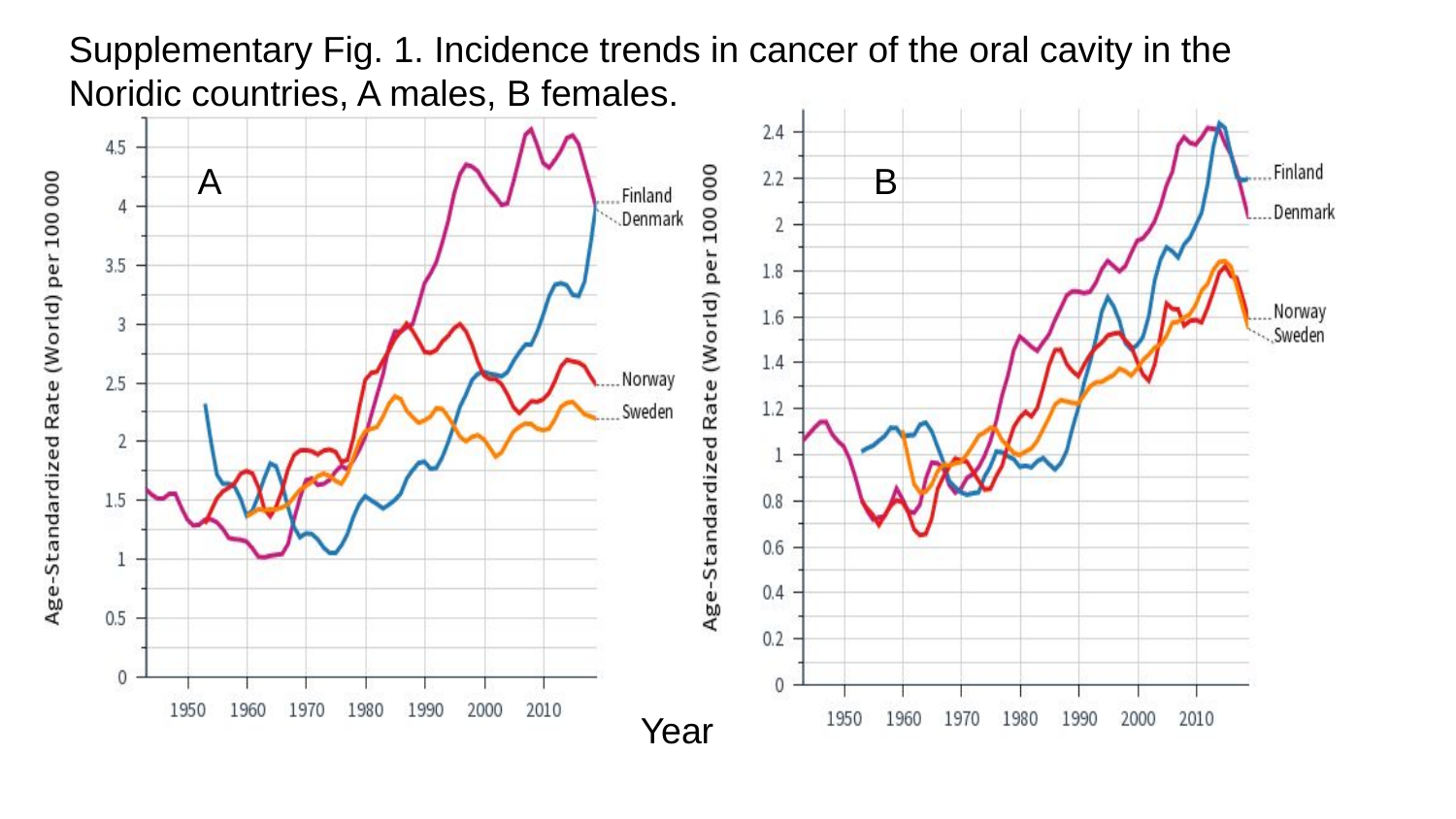

Supplementary Fig. 1. Incidence trends in cancer of the oral cavity in the Noridic countries, A males, B females.
A
B
Year
